# Retraction: MicroRNA-448 suppresses osteosarcoma cell proliferation and invasion through targeting EPHA7

**DOI:** 10.1371/journal.pone.0282985

**Published:** 2023-03-29

**Authors:** The PLOS ONE Editors

The *PLOS ONE* Editors retract this article [1] because it was identified as one of a series of submissions for which we have concerns about peer review, authorship, and similarities across articles. Image similarities were noted between the 0 hour scramble panel in Fig 3I in [1] and the 0h miR-599 panel of Fig 3 in [2]. These concerns call into question the validity and provenance of the reported results. We regret that the issues were not addressed prior to the article’s publication.

XW, YL, WX, LW, and XD either did not respond directly or could not be reached. LY responded but expressed neither agreement nor disagreement with the editorial decision.
